# Circular RNA MAP2K2‐modified immunosuppressive dendritic cells for preventing alloimmune rejection in organ transplantation

**DOI:** 10.1002/btm2.10615

**Published:** 2023-11-13

**Authors:** Shuailong Li, Amal Abu Omar, Adam Greasley, Bowen Wang, Tan Ze Wang, Serina Chahal, Raj Kumar Thapa, Douglas Quan, Anton Skaro, Kexiang Liu, Xiufen Zheng

**Affiliations:** ^1^ Department of Cardiovascular Surgery The Second Norman Bethune Hospital of Jilin University Changchun China; ^2^ Department of Pathology and Laboratory Medicine Western University London Ontario Canada; ^3^ Department of Surgery Western University London Ontario Canada; ^4^ Department of Microbiology and Immunology Oncology Western University London Ontario Canada; ^5^ Department of Oncology Western University London Ontario Canada; ^6^ Lawson Health Research Institute London Ontario Canada

**Keywords:** circMAP2K2, circular RNA, dendritic cell, immune rejection, immunosuppression, immunosuppressive DCs, organ transplantation

## Abstract

Long‐term patient and graft survival has been achieved in organ transplantation but at the expense of toxic side effects that are associated with long‐term use of nonspecific immunosuppressive drugs. Discovering new regulators of dendritic cells is the key for development of an ideal treatment to prevent immune rejection. We hypothesized that knockdown of circMAP2K2 induces immunosuppressive DCs and that treatment with circMAP2K2 silenced‐DCs can prevent alloimmune rejection. DCs were cultured and transfected with siRNA for circMAP2K2. circMAP2K2 levels were measured by qRT‐PCR. DC's maturation and immune function were assessed by flow cytometry and mixed lymphocyte reactions. The function of circMAP2K2 was illustrated by a series of RIP and IP. The therapeutics of engineered DCs was tested in a mouse heart transplantation model. We found that circMAP2K2 was highly expressed in mature DCs. Knockdown of circMAP2K2 reduced expression of MHCII, CD40 and CD80, attenuated the ability of DCs to activate allogeneic naïve T cells, and enhanced CD4^+^CD25^+^FOXP3^+^ regulatory T cells (Treg). circMAP2K2‐induced immunosuppressive DCs by interacting with SENP3. Treatment with circMAP2K2‐knockdown DCs attenuated alloimmune rejection and prolonged allograft survival in a murine heart transplantation model. The immune suppression induced in vivo was donor‐antigen specific. In conclusion, knockdown of circMAP2K2 can induce immunosuppressive DCs which are able to inhibit overactive immune response, highlighting a new promising therapeutic approach for immune disorder diseases.

**Translational Impact Statement**

This work demonstrates that immunosuppressive DCs can be generated by knocking down circMAP2K2 with siRNA and that treatment with circMAP2K2‐silenced DCs can induce donor‐specific immunosuppression, attenuate alloimmune rejection, and prolong transplant grafts in organ transplantation. circRNA‐modified immunosuppressive DCs hold promise in induction of donor‐antigen specific immune suppression and immune tolerance for organ transplantation or autoimmune diseases.

## 1 INTRODUCTION

Organ transplantation is only treatment option for end‐stage organ failure diseases. With the advancement of surgical technology, the survival rate of patients at 1 year after surgery is maintained at 80%–90%.^1^, ^2^, ^3^ However, the long‐term survival rate of transplant recipients has not been significantly improved due to immune rejection and side effects (toxicities, malignancy, and infection) resulting from long term immune suppression.^3^, ^4^, ^5^ How to tackle alloimmune rejection and induce donor antigen specific immunosuppression is the long‐term goal of research in organ transplantation. As the most powerful antigen presenting cells, bridging innate and adaptive immunity, dendritic cells (DCs) play a vital role in alloimmune rejection versus immune suppression in organ transplantation.^6^, ^7^, ^8^ Immunosuppressive dendritic cells induced by immune suppressive small molecules, overexpression of immune inhibitory genes, or knockdown of immune reactive genes, have been investigated for suppressing immune response.^7^, ^8^, ^9^, ^10^ However, new approaches to generate immunosuppressive DCs still need to be explored.

Recent studies have shown that circular RNA (circRNA), a single stranded RNA with closed loop structure, produced by alternative splicing known as a backsplicing event,^11^, ^12^, ^13^, ^14^ is a new type of regulator with multiple functions, and play vital roles in physiological and pathological processes.^7^, ^9^, ^11^, ^12^, ^15^ Interestingly, it has been reported circRNAs such as circRNAs from the Malat1, FSCN 1 and SNX5 genes are involved in DCs and modifying their expression results in engineered DCs as immunosuppressive for immune dysfunction diseases,^7^, ^9^, ^16^ suggesting that circRNA is a new regulator for DCs. More new circRNAs capable for regulating DC development and function need to be discovered and studied. The new approach for potent immunosuppressive DCs through modifying circRNA requires further investigation.

In this study, we aim to discover the role of circular RNA MAP2K2 (circMAP2K2), which is back‐spliced from the mitogen‐activated protein kinase 2 (MAP2K2, or MEK2) RNA and consists of exons 2, 3, and 4, in DCs and to investigate the potential of circMAP2K2 modified DCs on protection of transplant organs from immune rejection using a murine heart transplantation model.

## 2 MATERIALS AND METHODS

### 2.1 Animals

C57BL/6 and BALB/c 8‐week‐old wild‐type male mice were purchased from Charles River Laboratories (Charles River Laboratories Canada, Senneville, Quebec, Canada) and C3H/Hej male mice were purchased from the Jackson Laboratories (The Jackson Laboratories, Bar Harbor, Maine). All experimental animals were cared for following the guidelines set by the Canadian Council on Animal Care. All animal studies complied with the guidelines of the Animal Use Committee of Western University (AUP‐2019‐147).

### 2.2 DC culture, isolation and transfection with siRNA

Bone marrow‐derived DCs (BM‐DCs) were cultured from bone marrow progenitor cells of C57BL/6 mice with RPMI‐1640 medium (Thermo Fisher Scientific, Mississauga, Ontario, Canada) in the presence of 10% fetal bovine serum (FBS, Thermo Fisher Scientific), 10 ng/mL of granulocyte‐macrophage colony‐stimulating factor (GM‐CSF, PeproTech, Rochy Hill, NJ) and 10 ng/mL of interleukin 4 (IL‐4, PeproTech), and 100 U penicillin and streptomycin (Thermo Fisher Scientific), as described previously.^7^, ^9^, ^17^

On day 5 of cell culture, BM‐DCs (1 million cells/well in a 12‐well plate) were transfected with 1 μg siRNA using 2 μL EndoFectin™ (Gene Copoeia Inc., Rockville, MD) as described in Appendix S1.

### 2.3 DC staining and flow cytometry

DCs were collected and stained with fluorescent CD11c‐FITC, CD40‐PE‐cy5, CD80‐PE and MHCII‐fluor 450 (Biolegend, San Diego, CA), followed by flow cytometry analysis using a Cytoflex S (Beckman coulter, Indianapolis, IN) as described in Appendix S1. Mean fluorescence intensity (MFI) was collected and compared between groups.

To detect cell apoptosis and death, cells were stained with Annexin V‐APC and propidium iodide (PI) (Thermo Fisher), followed by flow cytometry following the manufacturer's instruction.

### 2.4 RNA isolation, qRT‐PCR, immunocytochemistry, and Western blotting

Total RNA was isolated from cells using TRIzol lysis reagents (ThermoFisher) following the instructions of the manufacturer. Concentration was measured at 260 nm using a DeNovix DS‐11 spectrophotometer (DeNovix, Wilmington, DE). qRT‐PCR were conducted using primers listed in Table S1.

Isolation of total Protein, and fractional isolation of cytosol/ nuclear protein, protein concentration measurement, Western blotting for MEK2, P‐p65, pERK1/2, SENP3, β‐actin and histone 3, and immunocytochemistry (ICC), were described in Appendix S1.

### 2.5 T cell labeling with violet cell‐tracer and mixed leukocyte reaction for T cell proliferation

Splenic cells were isolated from male BALB/c mice and lysed with ACK lysis buffer (Sigma) to removed red blood cells. After lysis, 10^7^ splenic T cells were labeled with Violet Cell‐Tracer (Thermo Fisher Scientific) for 15 min at 37°C according to the manufacturer's instruction.

Cell‐Tracer labeled T cells (2 × 10^5^ cells/well) were co‐cultured with DCs (4 × 10^4^ cells/well) in a U‐bottom 96 well plate with 5% CO_2_ at 37°C for 3 days. Cells were collected and stained with antibodies (Abs) against CD4 and CD8, followed by flow cytometry. Cell were first gated on CD4^+^ or CD8^+^ cells, and Cell‐Tracer intensity was measured and compared between groups.

### 2.6 Mitomycin C treatment and donor antigen T cell recall response

Splenic cells from donor‐strain C57BL/6 mice or a third party C3H mice were treated with 25 μg/mL Mitomycin C (Sigma) for 30 min at 37°C, followed by washing with PBS three times.

Splenic cells from transplant recipient BALB/c mice were isolated, labeled with violet cell‐tracer, and co‐cultured with the above mitomycin C treated splenic cells at the ratio of 5:1 for 72 h. Cells were collected and stained with fluorescent CD4, CD8 Abs, and subjected to flow cytometry to measure violet cell‐tracer dilution, which allowed us to assess T cell proliferation.^9^

### 2.7 Treg generation, staining and measurement

Treg generation in vitro was performed in a mixed leukocyte reaction where whole splenic T cells from naïve BALB/c mice were co‐cultured with DCs transfected with circMAP2K2 siRNA or GL2 siRNA at the ratio of 1:10 for 5–7 days.

Cells from co‐cultures in vitro or from in vivo heart transplant recipient animals were stained with CD4, CD25, and Foxp3 using a Treg staining kit (Thermo Fisher Scientific) following the manufacturer's instructions. Fluorescence intensity of CD4, CD25, and Foxp3 was measured by flow cytometry.

### 2.8 Biotinylated probe synthesis

Anti‐circMAP2K2 and random biotinylated probes were synthesized using DreamTaq DNA polymerase and Biotin‐14‐dCTPs (Thermo Fisher Scientific). Briefly, 0.02 μM probe oligo was mixed with 0.02 μM forward and 0.2 μM reverse primer in a standard DreamTaq reaction containing 5 mM of each dNTP. In this case, 2.5 mM dCTP was replaced by biotin‐14‐dCTP to allow 50% biotinylated dCTP in each probe. Probes were synthesized by 50 cycles of 95°C for 10 s, 42°C for 10 s, and 72°C for 10 s.

### 2.9 RNA in situ hybridization

Forty‐thousand DCs were plated on coverslips in a 24‐well plate with DC culture medium and incubated overnight. Cells were fixed with 4% paraformaldehyde, hybridized with biotinylated DNA probes complementary to the circRNA junction sequence, and visualized with FluorAlex 594‐stripavidin as described previously.^9^

### 2.10 RNA immunoprecipitation assays and immunoprecipitation

On day 8 of culture, DCs were treated with 100 ng/mL LPS for 2 h at 37°C, collected, and subjected to RNA immunoprecipitation (RIP) and Co‐immunoprecipitation (Co‐IP) as described in Appendix S1.

### 2.11 Mass spectrometry

Mass spectrometry (MS) was conducted on RIP immunoprecipitates pulled down using circMAP2K2 probes by Bioinformatic Solutions (Waterloo, ON, Canada) as described previously.^9^

### 2.12 Mouse heart transplantation

DCs (10^6^) transfected with circMAP2K2 siRNA or GL2 siRNA which targets firefly luciferase and was used as a control siRNA were injected into a BALB/c heart transplant recipient mice via the tail vein 7 days prior to surgery. A C57BL/6 mouse was used as donor of the heart. Heterotopic heart transplantation was conducted between C57BL/6 and BALB/c mice as described previously.^7^, ^9^ Post surgery, recipient mice were treated daily with rapamycin (1 mg/kg of body weight) for 7 consecutive days to inhibit acute rejection. Palpable heartbeat was blindly monitored twice a week, until the endpoint of experiments.

### 2.13 Hematoxylin and eosin or Masson's trichrome staining

At the endpoint of experiments, transplant hearts were collected from the recipient mice, subjected to hematoxylin and eosin (H&E) or trichrome staining, and scored blindly by pathologist for histopathological changes and fibrosis as described previously.^9^, ^18^, ^19^, ^20^

### 2.14 Statistical analysis

Data were presented as mean ± SD and graphs were prepared using GraphPad Prism 7.0. Unpaired or paired two‐sided *t*‐tests, and one‐way analysis of variance (ANOVA) were performed for comparison between two groups and among three groups, respectively. A log‐rank test was conducted for allograft survival data. The specific statistical analyses used are described in each figure legend. Differences with *p*‐values <0.05 were considered significant.

## 3 RESULTS

### 3.1 CircMAP2K2 level positively correlates to the maturity of DCs

**FIGURE 1:**
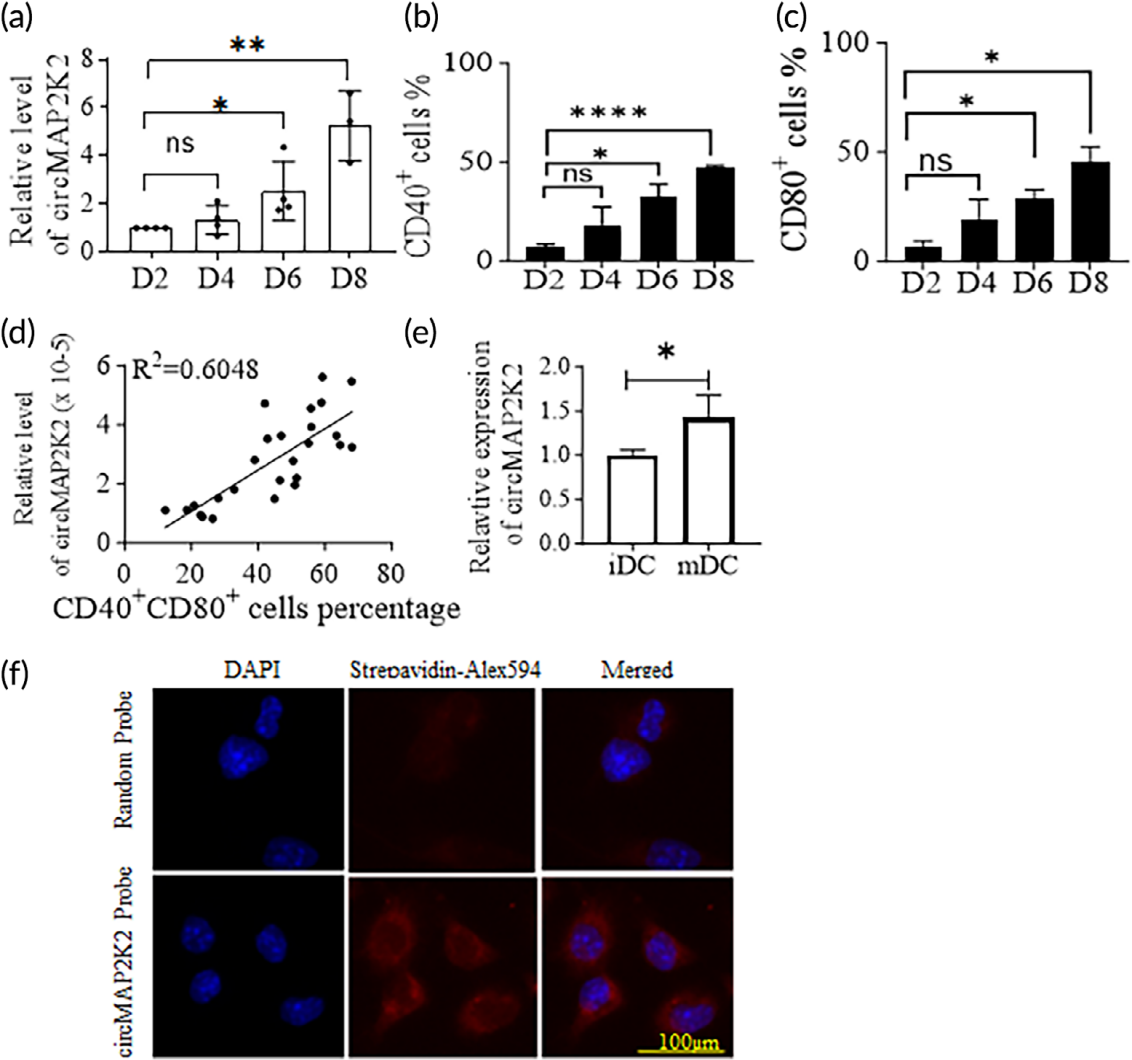
CircMAP2K2 expression level and cell distribution. (a) circMAP2K2 expression profile in DCs. BM‐DCs were cultured from C57BL/6 mice in vitro for measuring circMAP2K2 levels. Relative expression of circMAP2K2 in DCs at the indicated time points was measured by qRT‐PCR. (b, c) CD40 and CD80 expression. The above DCs were tested for the expression of CD40 and CD80 by flow cytometry. Cells were gated on live cells. (d) Correlation of relative circMAP2K2 and percentage of CD40^+^CD80^+^ cells. (e) circMAP2K2 upregulation in mDC. DCs were stimulated with LPS and circMAP2K2 expression was detected by qRT‐PCR. (f) In situ RNA hybridization. DCs were plated on glass coverslips, hybridized with biotin‐labeled circMAP2K2 DNA probes or random probes, then visualized with Alex 594‐streptavidin. Images were taken under a fluorescent microscope at the magnification ×200. Images were merged on Adobe Photoshop. Images are representative of three independent experiments. An unpaired t‐test was conducted, *n* = 3 per group, *n* = 27 for (d) and *n* = 4 for (e). *p < 0.05, **p < 0.01, and ****p < 0.0001.

To determine the relationship between circMAP2K2 levels and the maturity of DCs, bone marrow derived DCs (BM‐DCs) were cultured from C57BL/6 mice in the presence of IL‐4 and GM‐CSF in vitro. qRT‐PCR with divergent primers were conducted to measure circMAP2K2 expression. Flow cytometry was also performed to measure CD11c, CD40, and CD80 expression to determine the maturity of DCs. The time course experiments show that circMAP2K2 levels progressively rose as culture time increased (Figure 1a). The expression of CD40 and CD80 in CD11c^+^ cells showed the same trend as circMAP2K2 levels (Figure 1b,c). A correlation analysis shows that circMAP2K2 levels positively correlated with the percentage of CD40^+^CD80^+^ cells (Figure 1d). circMAP2K2 is up‐regulated in mature DCs (mDCs) compared with immature DCs (imDCs) (Figure 1e).

To determine cellular distribution of circMAP2K2 expression, in situ RNA hybridization was conducted with biotinylated probes that are complementary to the circMAP2K2 junction sequence and red fluorescent FluroAlex 594 conjugated Streptavidin. There was almost no or a very faint red fluorescence in the cells hybridized with random probes, whereas a very strong red fluorescence representing circMAP2K2 was observed in the cytoplasm of DCs hybridized with circMAP2K2 probes, not seen in the nucleus (Figure 1f).

### 3.2 Knockdown of circMAP2K2 reduces MHCII, and CD40, CD80, and CD86

**FIGURE 2:**
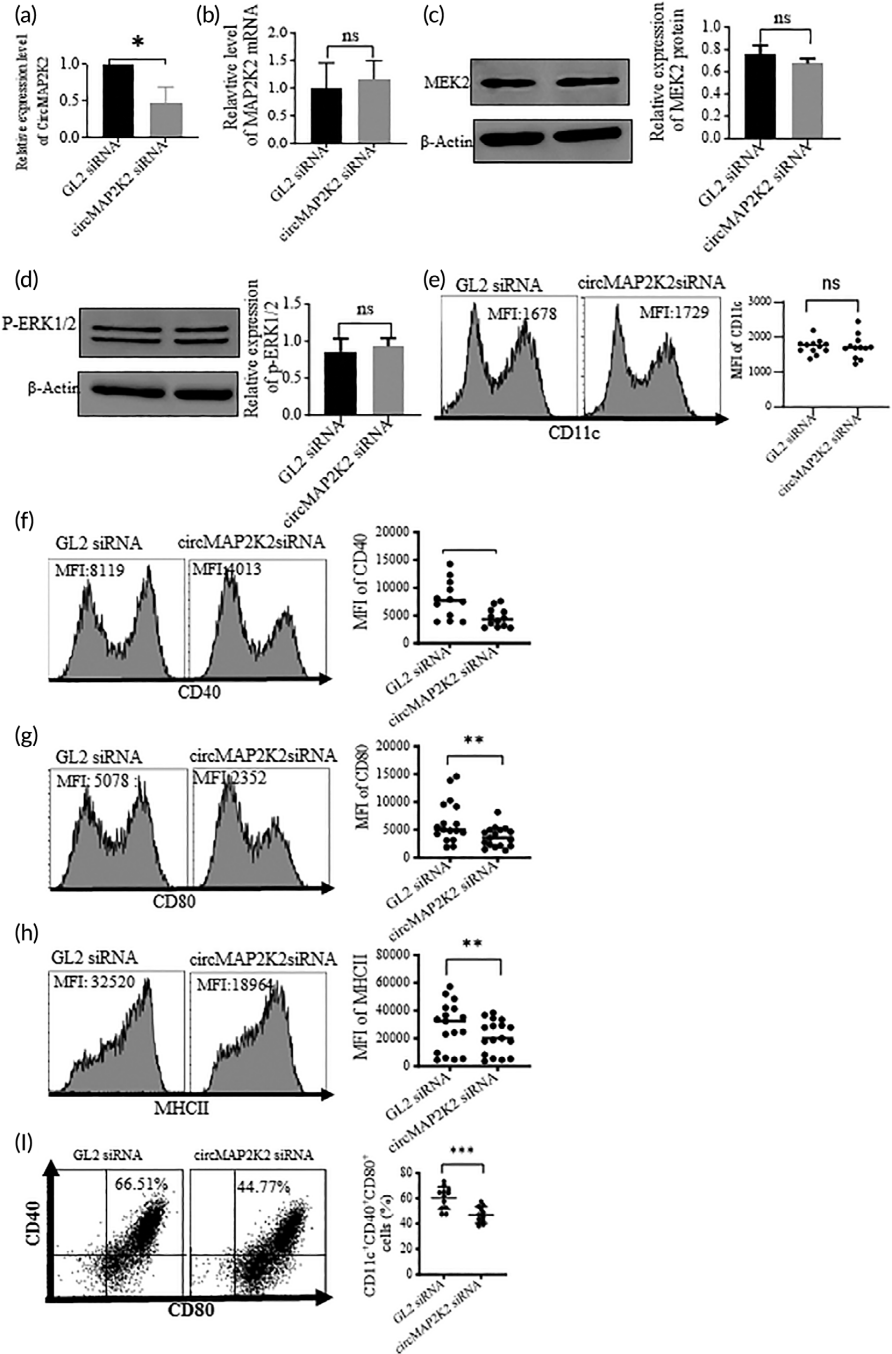
Knocking down circMAP2K2 in DCs with siRNA reduces MHCII, CD40, and CD80. (a) circMAP2K2 siRNA knocked down circMAP2K2. On day 5 of DC culture, DCs were transfected with circMAP2K2 siRNA or negative control (GL2) siRNA for 48 h. The relative expression of circMAP2K2 was measured by qRT‐PCR and normalized with GL2 siRNA. (b,c) circMAP2K2 siRNA did not affect MAP2K2/MEK2 mRNA level or protein. Forty‐eight hours after transfection, cells were subjected to RT‐PCR and Western blotting to measure the MEK mRNA (b) and protein (c), respectively. (d) Phosphorylated ERK1/2 expression. (e–h) Expression of DC surface markers CD11c, CD40, CD80, and MHC II. Forty‐eight hours after transfection, culture cells were stained with fluorescent Abs targeting CD11c, CD40, CD80, and MHC II, followed by flow cytometry. Cells were first gated on live cells, and MFI was measured. Left: Representative histogram graphs; Right: Summary data. (i) circMAP2K2 siRNA reduced CD40^+^CD80^+^ cells. *n* = 3 in (a)–(d) and *n* > 3 in (e)–(i). *p < 0.05; **p < 0.01; ***p < 0.001; ****p < 0.0001; ns, *p* > 0.05.

To investigate whether the expression of circMAP2K2 affects the development of DCs in vitro, we designed siRNA targeting the circMAP2K2 junction sequence and transfected them into DCs to knock down circMAP2K2. We found that transfection with circMAP2K2 siRNA decreased circMAP2K2 levels by 50% compared with control GL2 siRNA (Figure 2a), but did not significantly affect the expression of MAP2K2 /MEK2 at either the mRNA level (Figure 2b), or protein level (Figure 2c), 48 h post‐transfection. Knockdown of circMAP2K2 did not impact ERK1/2 phosphorylation (Figure 2d). There was no difference observed in cell apoptosis/death between circMAP2K2 siRNA and GL2 siRNA (Figure S1). The expression of CD11c did not change after circMAP2K2 knockdown, measured by flow cytometry (Figure 2e). In contrast, circMAP2K2 siRNA significantly reduced the expression of MHCII, CD40, and CD80 with much lower mean fluorescent intensity (MFI) compared to GL2 siRNA (Figure 2f–h). Furthermore, the percentage of CD40^+^CD80^+^ DCs was significantly lower after transfection with circMAP2K2 siRNA compared with GL2 siRNA (Figure 2i).

### 3.3 Knockdown of CircMAP2K2 inactivates the NFKB pathway and reduces the expression of pro‐inflammatory cytokine genes and CCR7

**FIGURE 3:**
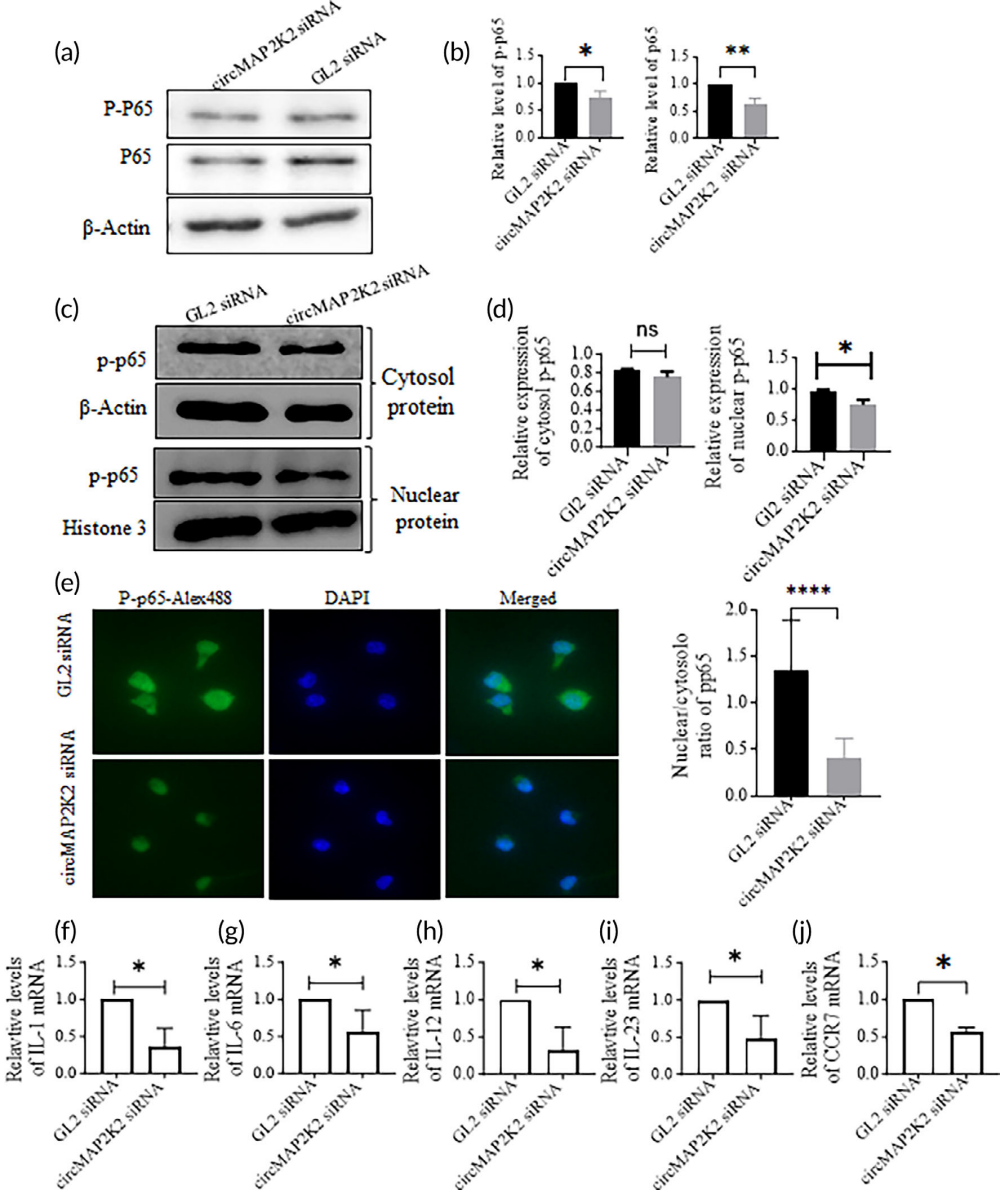
Knockdown of circMAP2K2 lowers the expression of phosphorylated P65, pro‐inflammatory cytokines and CCR7. (a) Representative Western blotting images of phosphorylated P65. DCs transfected with circMAP2K2 siRNA or GL2 siRNA for 48 h were subjected to Western blotting with Abs against p‐p65, and p65. (b) Relative levels of p‐p65 and p65 proteins. Densities of Western blotting bands were semi‐quantitatively measured by Image J, relative protein levels were normalized with control GL2 siRNA transfected cells, β‐actin was used as a loading control. (c, d) Cytosol and nuclear p‐p65 protein expression. DCs were treated as (a), harvested and then subjected to fractional isolation for cytosol and nuclear protein, followed by Western blotting. Cytosol p‐p65 was normalized with β‐actin, nuclear p‐65 was normalized with histone 3. (c) Representative images; (d) summary data. (e) Immunocytochemistry (ICC) for p‐p65. ICC was conducted on siRNA transfected cells 48 h after transfection and imaged under a fluorescent microscope at the magnification ×200 and merged by Adobe Photoshop. Green: P‐p65: Blue: DAPI. Left: Representative images; Right: Ratio of nuclear/ cytosol p‐p65. Fluorescent of p‐p65 in the nuclei and cytoplasm respectively were measured using Image J. *n* (cell number) = 30. (f–j) Relative mRNA levels of IL‐1, IL‐6, IL‐12, IL‐23, and CCR7. Relative mRNA levels of IL‐1, IL‐6, IL‐12, IL‐23, and CCR7 were measured by qRT‐PCR and normalized with GL2 siRNA. β‐Actin was used as a loading control. Representative images were selected from three independent experiments. Unpaired t test was conducted. *n* = 3. *p < 0.05; **p < 0.01; ***p < 0.001; ****p < 0.0001; ns, *p* > 0.05.

Western blotting was executed on DCs ransfected with circMAP2K2 siRNA or GL2 siRNA using Abs targeting phosphorylated P65 (P‐p65). The results show that knockdown of circMAP2K2 reduced p‐p65 protein (Figure 3a,b). We then fractionally isolated cytosol and nuclear proteins, followed by Western blotting. It was found that both cytosol and nuclear P‐p65 were lower in circMAP2K2 siRNA transfected group than control GL2 siRNA group (Figure 3c,d), and the difference in nuclear P‐p65 was significant. We also conducted ICC to confirm that circMAP2k2 siRNA inhibited P‐p65 nuclear translocation (Figure 3e).

Moreover, we found that transfection of circMAP2K2 siRNA significantly reduced the expression of IL‐1, IL‐6, IL‐12, IL‐23, and chemokine receptor CCR7 as compared with GL2 siRNA (Figure 3f–j).

### 3.4 Knockdown of circMAP2K2 reduces DCs to activate allogeneic T cells and enhances DCs to generate Tregs

**FIGURE 4:**
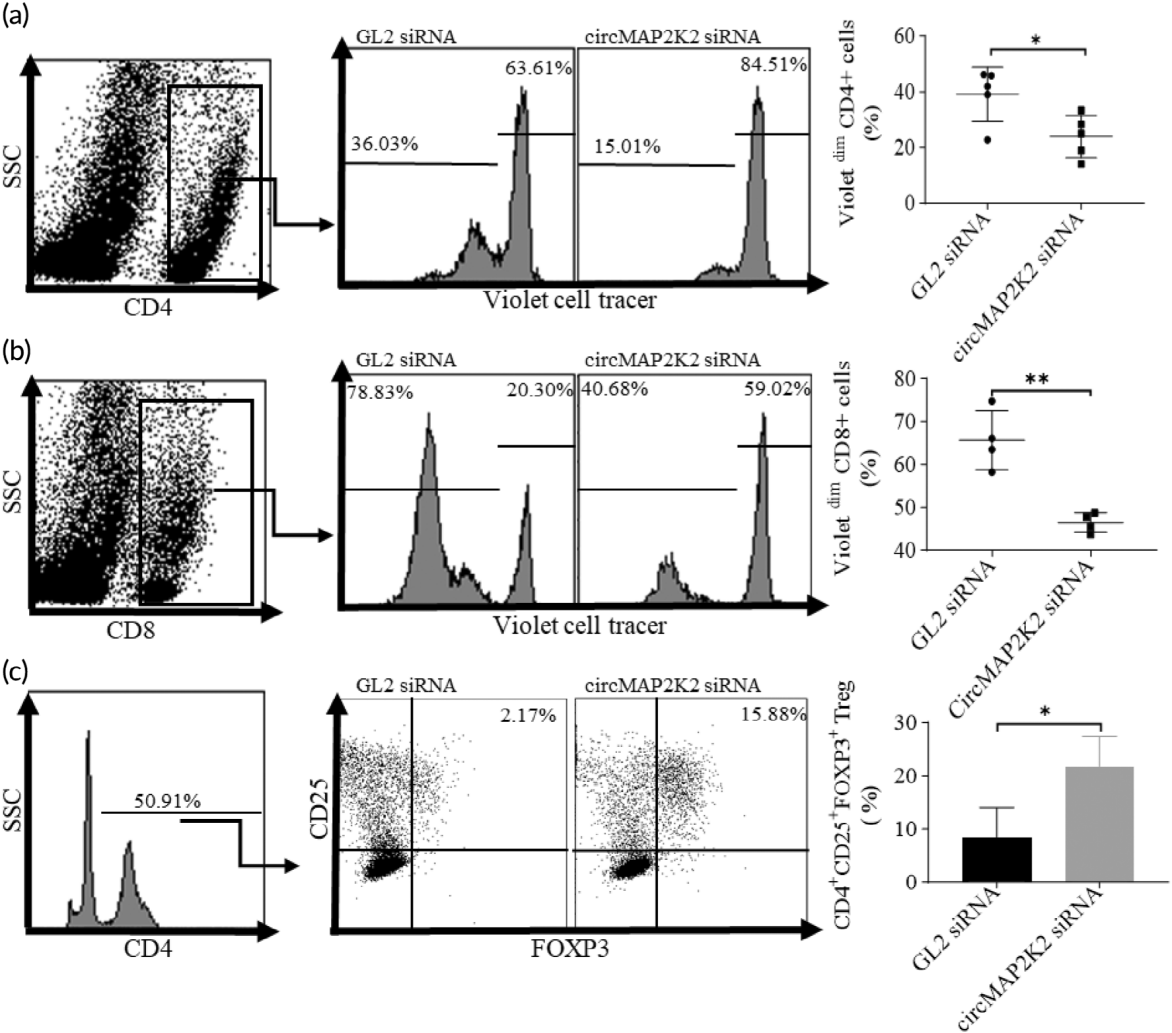
Knockdown of circMAP2K2 decreases DC's ability to activate naïve T cells but enhances the generation of CD4^+^CD25^+^Foxp3^+^Tregs. (a, b) Knockdown of circMAP2K2 decreases DC's ability to activate naïve T cells. siRNA transfected Day 7 DCs from C57BL/6 mice were co‐cultured with violet cell tracer labeled allogeneic naïve T cells from BALB/c mice at the ratio of DCs:T cells = 1:5 for 72 h. Cells were stained with CD4‐PE and CD8 PC5.5 Abs and measured by flow cytometry. Cells were gated on CD4^+^ T cells (A) and CD8^+^ T cells (b). Violet^dim^ cells represent proliferative cells while Violet^high^ represents non‐proliferative cells. (c) Silencing circMAP2K2 increases the generation of CD4^+^CD25^+^Foxp3^+^Tregs. siRNA transfected Day 7 DCs were co‐cultured with allogeneic naïve T cells at the ratio of DCs:T cells = 1:10 for 7 days. Cells were stained with CD4‐FITC, CD25‐PC7 and Foxp3‐PE Abs, the intensity of fluorescence was measured by flow cytometry. Cells were gated on viable CD4^+^ cells. Unpaired *t*‐test was conducted. *n* = 4 for (a) and (b), *n* = 3 for (c). *p < 0.05, **p < 0.01.

To determine whether knockdown of circMAP2K2 alters DC's capacity to activate T cells, we conducted mixed leukocyte reactions (MLRs) where circMAP2K2‐siRNA transfected DCs were co‐cultured with violet cell‐tracer labeled naïve allogeneic T cells from BALB/c mice. The dilution of cell‐tracer was measured by flow cytometry to determine T cell proliferation after co‐culture. To discriminate between CD4 and CD8 T cells within the proliferated T cell subset, cultured cells were stained with different fluorescent CD4 and CD8 Abs prior to flow cytometry. Fewer cell‐tracer ^dim^CD4^+^ T cells, which were recognized as proliferative cells, were detected in the culture with circMAP2K2‐silenced DCs than those co‐cultured with GL2 siRNA DCs (Figure 4a), indicating that circMAP2K2 siRNA reduces DCs to activate CD4^+^ T cells. A similar result was observed with CD8^+^ T cells, where they proliferated at a lower rate after the co‐culture with circMAP2K2‐silenced DCs compared with GL2‐DCs (Figure 4b).

To study whether knockdown of circMAP2K2 in DCs increases Tregs, circMAP2K2‐siRNA transfected DCs were cultured with allogeneic naïve T cells in vitro, followed by staining with Abs against CD4, CD25 and Foxp3, and detection via flow cytometry. As shown in Figure 4c, the percentage of CD4^+^CD25^+^Foxp3^+^ Tregs in the circMAP2K2‐knockdown DCs group was significantly higher than in the control group.

### 3.5 CircMAP2K2 binds to SENP3 and regulate SENP3 levels in DCs

**FIGURE 5:**
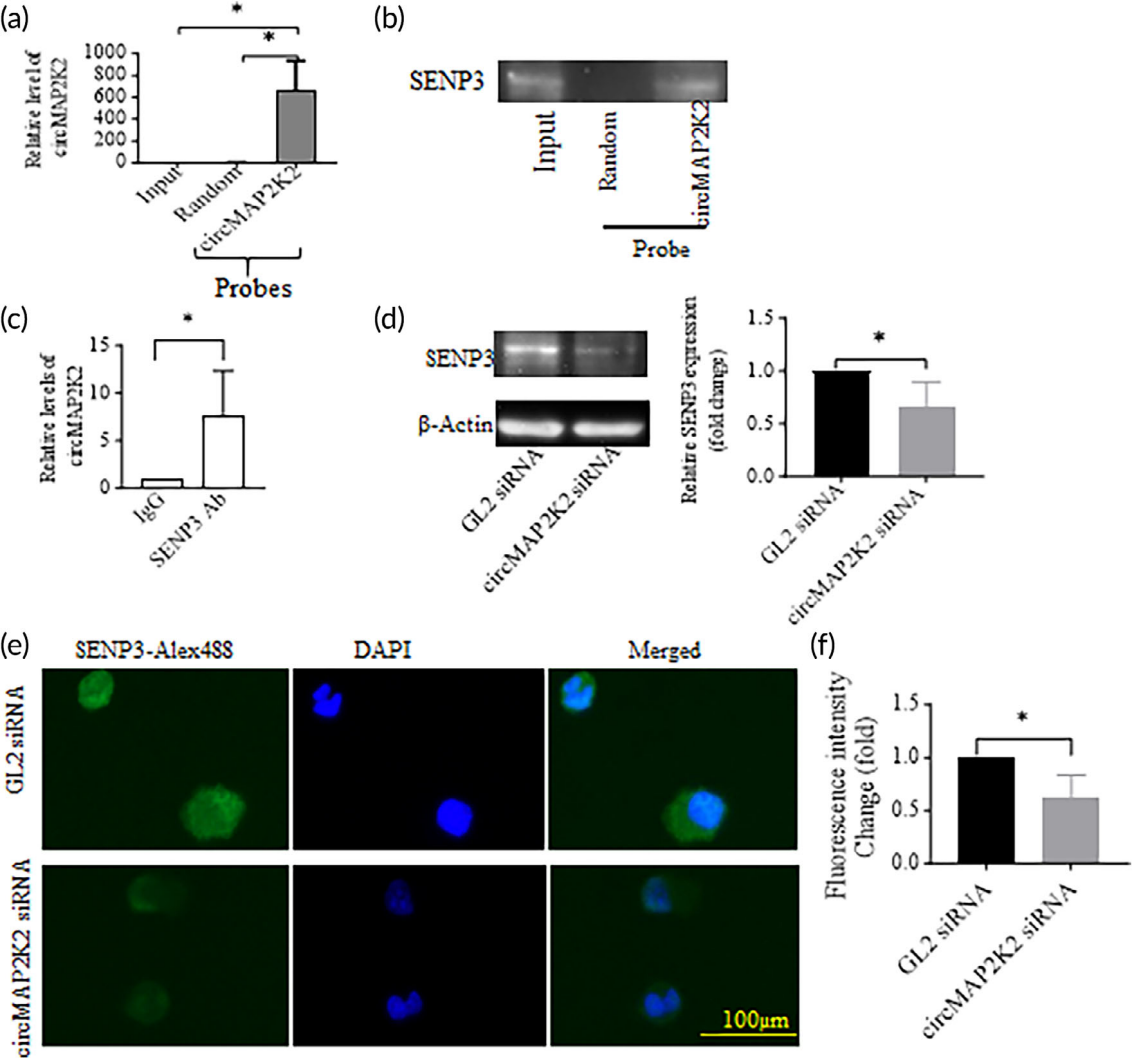
circMAP2K2 binds to SENP3. (a) RIP assays with circMAP2K2 probes. Cell lysates from DCs cultured for 7 days were incubated with biotinylated circMAP2K2 DNA probes or random probes, followed by qRT‐PCR for circMAP2K2 RNA levels. (b) circMAP2K2 probes pulled down SENP3. RIP assays with circMAP2K2 probes were conducted as described in (a), followed by Western blotting for SENP3. Representative images from three independent experiments. (c) IP assays for circMAP2K2. Cell lysates from DCs cultured for 8 days were incubated with SENP3 Abs or IgG and then with Sepharose A/G beads. qRT‐PCR was conducted to measure pull‐down circMAP2K2 levels. (d) circMAP2k2 knockdown reduced SENP3. Forty‐eight hours after transfection with siRNA, cells were collected for measuring SENP3 expression by Western blotting. (e) Representative images of immunohistochemistry of SENP3. CircMAP2K2 siRNA or GL2 siRNA transfected DCs were plated on glass coverslips overnight, followed by ICC with SENP3 Abs, and FluorAlex 488 labeled second Abs. DAPI was used to stain the nucleus. Images were taken under a fluorescent microscope at the magnification ×200. Images were merged using Image J. (f) Total fluorescent intensity changes of SENP3. Fluorescent intensity of SENP3 were measured by Image J. Thirty individual cells from three independent experiments were measured. *p < 0.05.

To understand how circMAP2K2 functions in DCs, RIP assays were conducted on DC lysate using a biotin‐labeled probe that targets the circMAP2K2 junction sequence and a random probe control. The pulldown was subjected to RNA isolation and qRT‐PCR for circMAP2K2. It was found that circMAP2K2 was enriched by the circMAP2K2 probe rather than the random control probe (Figure 5a), indicating circMAP2K2 probes are able to pull down circMAP2K2.

Next, MS was conducted on the pulldown by circMAP2K2 probes to screen proteins bound by circMAP2K2. The MS results show that the circMAP2K2 probes captured SUMO‐specific protease 3 (SENP3) 3‐fold more than the random probes. To confirm MS results, Western blotting was conducted on RIP immunoprecipitates. The results show that more SENP3 protein were effectively pulled down by circMAP2K2 probes (Figure 5b).

To further validate the interaction of circMAP2K2 and SENP3, IP assays were conducted with SENP3 Abs, or IgG control. Precipitates were subjected to qRT‐PCR for circMAP2K2. The results show that circMAP2K2 was enriched by SENP3 Abs not by IgG (Figure 5c).

To further determine the relation of circMAP2K2 and SENP3, Western blotting was conducted on DCs transfected with circMP2K2 siRNA and GL2 siRNA transfected DCs. As shown in Figure 5d, knockdown of circMAP2K2 reduced the expression of SENP3.

ICC was also conducted to determine expression of SENP3 and their sub‐cellular distribution. It was found SENP3 were expressed in both the cytoplasm and nucleus. The total green fluorescent intensities for proteins SENP3 were significantly lower in circMAP2K2 siRNA transfected DCs than the GL2 control siRNA transfected cells (Figure 5e,f), which is consistent with the Western blotting results.

### 3.6 Treatment with circMAP2K2 knockdown DCs in vivo prolongs allograft survival and induces donor antigen specific immunosuppression

**FIGURE 6:**
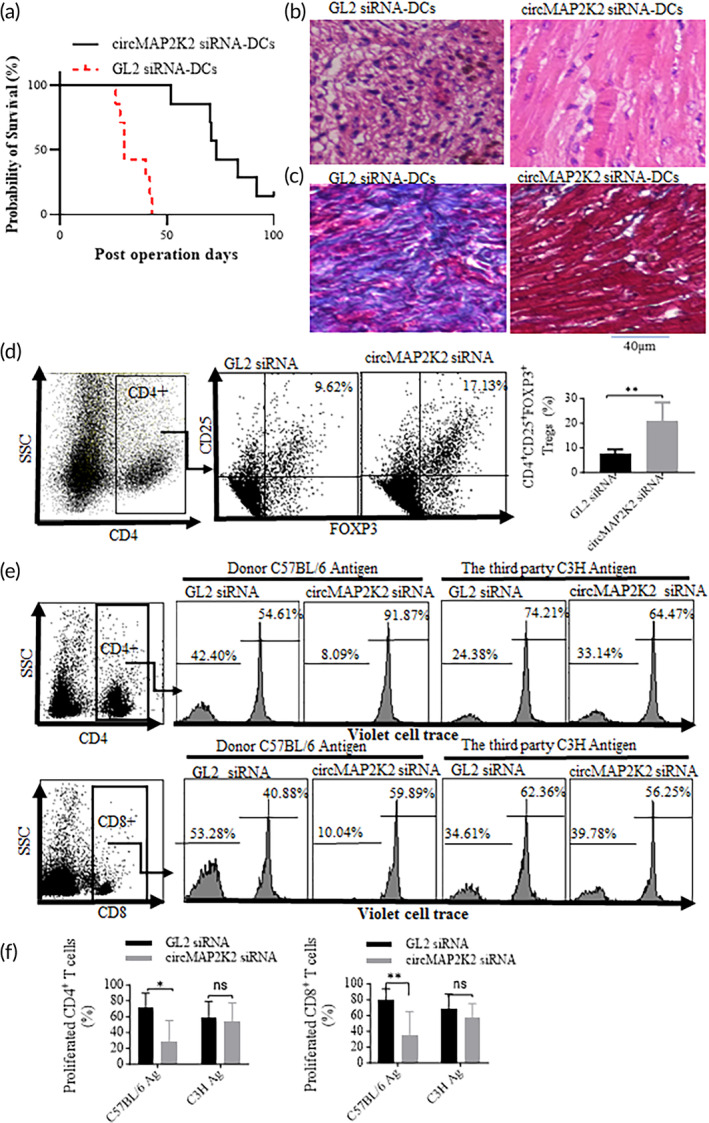
Treatment with circMAP2K2 knockdown DCs prolongs graft survival and induces donor antigen specific immune suppression. (a) Allograft survival. In vitro cultured C57/B6 mouse bone marrow‐derived CircMAP2K2 knockdown or negative control DCs (1 M cells per mouse) were injected into recipient BALB/C mice via i.v. injection through the tail vein. 7 days after injection, a MHC full‐mismatch heart transplantation was conducted between a C57/B6 donor mouse and BALB/C recipient mouse. Rapamycin (1 mg/kg per day) was i.p. injected for 7 days after surgery. *n* = 7. **p < 0.001. (b, c) H&E and Masson's trichrome staining. Grafts from circMAP2K2 knockdown and GL2 siRNA BALB/C recipient mice were collected at the endpoint of the experiment and subjected to H&E (b) and Masson‘s trichrome staining (c) for heart graft injury and fibrosis, respectively. (d) Tregs in vivo. Splenic cells from recipient mice were subjected for CD4^+^CD25^+^Foxp3^+^ cell staining, followed by flow cytometry. (e, f) Donor antigen specific suppression of T cells. The splenic cells of the recipient BALB/C mice isolated at the endpoint and labeled with violet cell‐tracer were co‐cultured with the mitomycin‐treated splenic cells of naïve C57/B6 or C3H mice for 72 h. T cell proliferation was measured by flow cytometry, following staining cells with CD4‐PE and CD8 Pe‐PC5.5 Abs. (e) Representative flow graphs; (f) quantitative results. GL2 siRNA‐DC group, *n* = 5, circMAP2K2 siRNA‐DC group, *n* = 7, **p < 0.01.

To test whether engineered immunosuppressive DCs by circMAP2K2‐knockdown can mitigate alloimmune rejection in organ transplantation, BM‐DCs were cultured from C57BL/6 mice, transfected with siRNA, and i.v. injected into recipient BALB/c mice (1 million of DCs/animal). Seven days after DC injection, and a mouse MHC mismatched heterotopic heart transplantation was conducted where the heart from a C57BL/6 donor mouse was implanted into the abdomen of a DC‐pre‐injected BALB/C recipient mouse. Palpable heartbeat was monitored in a double‐blind manner and immune rejection was claimed when heartbeat stopped. The results showed an average survival time of 40 days for transplant hearts in the control GL2 siRNA‐transfected DCs group and, in contrast, 73 days for those in the circMAP2K2 knockdown‐DC treatment group (Figure 6a). At the endpoint, transplanted hearts were collected for H&E staining and trichrome staining. The H&E results (Figure 6b) showed that cell infiltration and degeneration were observed in all transplant hearts. Myocardium in the heart grafts from the control DC group was completed destroyed without any normal structure. In contrast, typical structure of myocardium was still seen in the circMAP2K2 knockdown DC group. Similar results were seen in cell infiltration. Trichrome staining results show that there was massive fibrosis across entire grafts of control GL2 siRNA DCs treated groups, whereas fibrosis was only seen in the epicardium and not in the myocardium in the circMAP2K2 knockdown DC group (Figure 6c).

Moreover, the percentage of CD4^+^CD25^+^Foxp3^+^ Treg cells was increased in circMAP2K2 knockdown DC group compared with the control GL2 siRNA‐DCs treatment group (Figure 6d).

To verify whether the immunosuppressive effect is donor‐antigen specific, at the endpoint, we isolated splenic cells from recipient mice and cultured them with mitomycin C‐treated splenic cells from donor strain C57BL/6 mice, or from C3H mice which were used as the third party antigen. These mitomycin C‐treated splenic cells were used as donor‐derived antigen and the third party antigen, respectively. The results showed that T cells isolated from the control group recipient mice proliferated at a faster rate than those from the circMAP2K2 silenced DC group, in response to C57BL/6 derived antigen (Figure 6e,f). In contrast, there was no significant difference in T cell proliferation between the two groups, in response to C3H derived antigens (Figure 6e,f).

## 4 DISCUSSION

In this study, for the first time, we discovered that circMAP2K2 is highly expressed in mature DCs, knockdown of circMAP2K2 with siRNA induces immunosuppressive DCs, and treatment with circMAP2K2‐knockdown immunosuppressive DCs attenuates alloimmune rejection, prolonging transplant graft survival in a mouse heart transplant model. We provided a new approach to generate immunosuppressive DCs which can be used to treatment immune disorder diseases and suppress immune system reaction.

DCs originate from bone marrow progenitor cells and differentiate into immature DCs and mature DCs. In vitro, DCs can be cultured and differentiated from either bone marrow progenitor or monocytes from peripheral blood. Many molecules and signal pathways participate in these developmental and differentiation processes. circRNAs have recently been reported to be associated with DCs,^7^, ^9^, ^21^ but it is still understudied in DCs. There are no reports about circMAP2K2 except its expression reported by RNAseq.^22^ Here, we discovered that circMAP2K2 is over‐expressed in mDCs. Its expression levels steadily rise as DCs mature in in vitro culture and positively correlates with the expression levels of CD40 and CD80 molecules. CircMAP2K2 was majorly distributed in the cytoplasm, agreeing with that exonic circRNA is predominately expressed in the cytoplasm.^11^, ^12^, ^13^, ^14^

This study shows that circMAP2K2 siRNA reduced circMAP2K2 levels, but did not affect MEK mRNA or protein levels. Phosphorylation of ERK which is a downstream molecule of MEK 2 was not influenced by circMAP2K2 knockdown. These data suggest that circMAP2K2 may not affect MEK/ERK signaling. We also found that silencing of circMAP2K2 did not change the expression of CD11c, indicating circMAP2K2 does not affect DC differentiation from bone marrow progenitors. Interestingly, we found that knockdown of circMAP2K2 reduced the expression of MHCII, CD40, and CD80 in DCs, suggesting that silencing of circMAP2K2 reduces the maturity of DCs, retaining them as imDCs. We further found that knockdown of circMAP2K2 reduced the phosphorylation of p65, decreasing nuclear p‐p65. p65 is a family member of the NF‐κB pathway, which is important for DC maturation^23^ and is responsible for the transcription of pro‐inflammatory genes, which aid with inflammatory T cell activation.^24^, ^25^ Moreover, we observed circMAP2K2 knockdown reduces the expression of cytokines IL‐1, IL‐6, IL‐12, IL‐23 and chemokine receptor CCR7 in DCs. These cytokines are the third signal for DC to activate naïve T cells and are transcribed by the NFKB pathway. The data further confirm that circMAP2K2 down‐regulation results in the production of immunosuppressive imDCs and circMAP2K2 impacts NFKB signaling.

**FIGURE 7:**
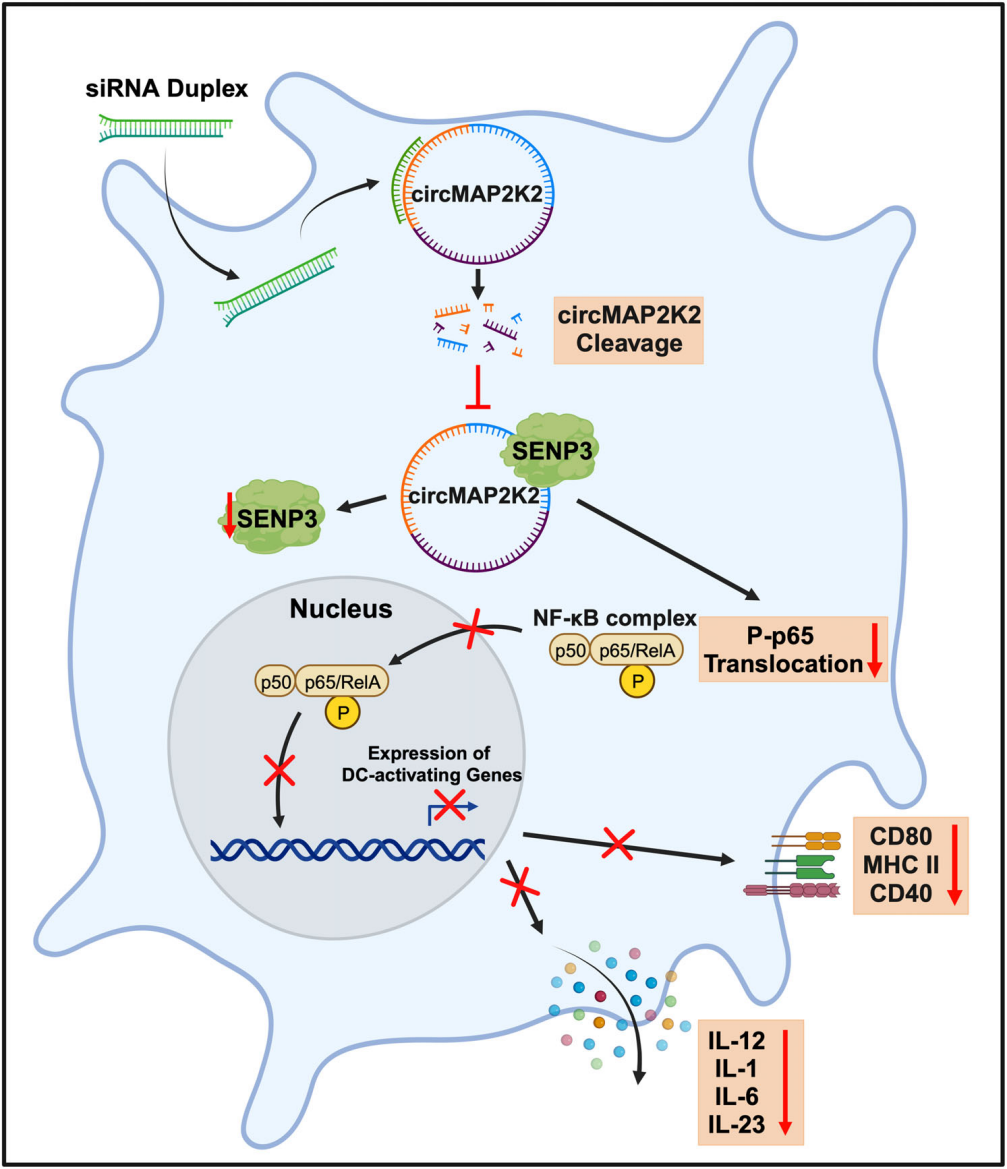
Schematic overview of the role of circMAP2K2 in regulation of DCs. CircMAP2K2 expressed in DCs binds to and stabilizes SENP3, leading to increased SENP3 protein expression and nuclear translocation of the canonical NF‐κB pathway dimers, P‐p65/p50 through an unknown intermediator(s). Increased P‐p65/p50 nuclear translocation results in increased transcription of NF‐κB‐regulated, DC‐activating genes, leading to the production of inflammatory, mature DCs, described phenotypically as MHC II^hi^CD40^hi^CD80^hi^ DCs and by the expression of pro‐inflammatory cytokines (IL‐1, IL‐6, IL‐12, and IL‐23). Specific knockdown of circMAP2K2 using siRNA that targets the junction sequence of circMAP2K2 results in the reduction of both SENP3 and P‐p65/p50 nuclear translocation, promoting the induction of immunosuppressive DCs, characterized as MHC II^lo^CD40^lo^CD80^lo^ DCs and through reduced expression of pro‐inflammatory cytokines; together these two characteristics result in a hypoimmune DC response. (graphic created with BioRender.com).

circRNA could function through interaction with proteins.^9^, ^11^, ^18^ In this study, we found that circMAP2K2 bound to SENP3 protein. SENP3 is a SUMO2/3‐specific protease and preferably binds to SUMO2/3.^26^ The physiological roles of SENP3 in DC development and function remain largely unclear. Nonetheless, it has been reported deficiency of SENP3 in DCs reduces interferon responsive genes and DC maturation related genes such as CD86, suggesting that SENP3 expression is helpful for DC's immune response.^27^ Our results show that knockdown of cirMAP2K2 reduced SENP3 expression and inhibited the activation of the NF‐κB pathway, resulting in decreased inflammatory cytokines. Therefore, these data suggest that circMAP2K2 binds to SENP3, regulating SENP3 and p‐p65, consequently, modulating DC development (Figure 7).

DCs are unique immune cells capable of activating naïve T cells, but require co‐stimulatory molecules to provide a secondary signal of activation to T cells. Reduction in co‐stimulatory molecules impairs DCs' ability to activate naïve T cells, conversely causing T cell inactivation.^28^ Our MLR results for T cell proliferation show that the proliferation of CD4^+^ and CD8^+^ T cells were significantly decreased when they were co‐cultured with circMAP2K2‐knockdown DCs. This result further demonstrated that circMAP2K2 knockdown‐DCs are immunosuppressive, failing to activate T cells properly. This defect in immune function of circMAP2K2 knockdown‐DCs is compatible with the results of reduced co‐stimulatory molecules, maturity and pro‐inflammatory cytokine expression.

Tregs are critical to immune tolerance^29^ and promotion of Treg generation is one of most important functions of immunosuppressive DCs.^30^, ^31^ In the current study, we demonstrated that knockdown of circMAP2K2 increases CD4^+^CD25^+^Foxp3^+^ Tregs both in vitro and in vivo, indicating these DCs are associated with immunosuppression or immune tolerance and they could be used to treat immune overeactive diseases such as organ transplantation and autoimmune diseases.

In past preclinical studies, in vitro‐prepared immunosuppressive imDCs have been introduced to prevent alloimmune rejection in organ transplantation^7^, ^8^, ^9^ and autoimmune diseases.^32^, ^33^, ^34^ In the current study, we show that administration of a single dose of circMAP2K2‐knockdown immunosuppressive DCs significantly prolonged allograft survival compared to those control DCs. We also demonstrated that the induced immunosuppression was donor‐antigen specific, avoiding infection‐ and malignancy‐related morbidity and mortality. Taken together, circAMP2K2 modified immunosuppressive DCs can direct immune response towards immune suppression in vivo, alloimmune rejection in organ transplantation is attenuated, and long‐term transplant graft survival is achievable with such new DCs therapy.

In summary, this is the first report on the role circMAP2K2 in DC‐mediated immune modulation and a new approach to induce immunosuppressive DCs through controlling circMAP2K2 expression. We demonstrated that circMAP2K2 level positively correlates to maturity of DCs. We demonstrated that knockdown of circMAP2K2 generates immunosuppressive DCs and that treatment with circMAP2K2‐knockdown DCs induces donor‐specific immunosuppression, attenuates alloimmune rejection and prolongs transplant graft in organ transplantation. This study enhances our understanding on the role circRNAs play within DCs. It also provides new insight into a new strategy for induction of immunosuppressive DCs and development of a new treatment to prevent transplant immune rejection and autoimmune diseases.

## AUTHOR CONTRIBUTIONS

**Shuailong Li:** Formal analysis (lead); investigation (lead); methodology (lead); visualization (lead); writing – original draft (lead); writing – review and editing (equal). **Amal Abu Omar:** Investigation (supporting); validation (supporting); visualization (supporting); writing – review and editing (supporting). **Adam Greasley:** Investigation (supporting); validation (supporting); writing – review and editing (supporting). **Bowen Wang:** Investigation (supporting); methodology (supporting); writing – review and editing (supporting). **Tan Ze Wang:** Investigation (supporting); validation (supporting); writing – review and editing (supporting). **Serina Chahal:** Investigation (supporting); validation (supporting); visualization (supporting); writing – review and editing (equal). **Raj Kumar Thapa:** Formal analysis (equal); investigation (supporting); validation (supporting); writing – review and editing (supporting). **Douglas Quan:** Conceptualization (supporting); writing – review and editing (supporting). **Anton Skaro:** Conceptualization (supporting); writing – review and editing (supporting). **Kexiang Liu:** Conceptualization (equal); funding acquisition (supporting); supervision (equal); writing – review and editing (supporting). **Xiufen Zheng:** Conceptualization (lead); funding acquisition (lead); project administration (lead); resources (lead); supervision (lead); writing – original draft (equal); writing – review and editing (equal).

## FUNDING INFORMATION

The study was supported by grants provided by the Canadian Institutes of Health Research (MOP#142278 and PJT 162448), the Natural Sciences and Engineering Research Council of Canada (RGPIN‐2019‐04545) to Xiufen Zheng, and the Chinese Scholarship Council to Shuailong Li (202006170211).

## CONFLICT OF INTEREST STATEMENT

The authors of this manuscript have no conflicts of interest to disclose as described by Bioengineering & Translational Medicine.

## Supporting information


**TABLE S1.** Primer, probe and siRNA sequences.
**FIGURE S1.** circMAP2K2 siRNA did not change cell apoptosis/death. In vitro cultured DCs were transfected with circMAP2K2 siRNA or control GL2 siRNA for 48 h. Cells were then collected and stained with fluorescent APC‐Annexin V and PI (Therom Fisher Scientific) according to the manufacturer's instruction. The intensity of fluorescence was measured using a Cytoflex S (Beckman). Left: Representative graphs of dot plot results; Right: Summarized data of Annexin V positive cell percentage.
**FIGURE S2.** Knockdown of circMAP2K2 in DCS enhanced CD4+ CD25+ Foxp3+ cell production. CD4^+^CD25^−^ T cells were isolated from naïve Babl/c mice using a CD4CD25 T cell isolation kit ((Miltenyi Biotec, San Jose, CA) following the manufacturer's instruction. Isolated CD4^+^CD25^−^ T cells were co‐cultured with circMAP2K2 siRNA or GL2 siRNA transfected DCs at the ratio of 1:10 for 5–7 days.

## Data Availability

Data available on request from the authors.

## References

[1] Khush KK , Potena L , Cherikh WS , et al. The international thoracic organ transplant registry of the International Society for Heart and Lung Transplantation: 37th adult heart transplantation report‐2020; focus on deceased donor characteristics. J Heart Lung Transplant. 2020;39(10):1003‐1015. 32811772 10.1016/j.healun.2020.07.010 PMC7737223

[2] Poggio ED , Augustine JJ , Arrigain S , Brennan DC , Schold JD . Long‐term kidney transplant graft survival‐making progress when most needed. Am J Transplant. 2021;21(8):2824‐2832. 33346917 10.1111/ajt.16463

[3] Reul RM Jr , Zhang TS , Rana AA , Rosengart TK , Goss JA . Consistent improvements in short‐ and long‐term survival following heart transplantation over the past three decades. Clin Transplant. 2021;35(4):e14241. 33524177 10.1111/ctr.14241

[4] Lodhi SA , Lamb KE , Meier‐Kriesche HU . Solid organ allograft survival improvement in the United States: the long‐term does not mirror the dramatic short‐term success. Am J Transplant. 2011;11(6):1226‐1235. 21564524 10.1111/j.1600-6143.2011.03539.x

[5] Wilhelm MJ . Long‐term outcome following heart transplantation: current perspective. J Thorac Dis. 2015;7(3):549‐551. 25922738 10.3978/j.issn.2072-1439.2015.01.46 PMC4387387

[6] Zhuang Q , Liu Q , Divito SJ , et al. Graft‐infiltrating host dendritic cells play a key role in organ transplant rejection. Nat Commun. 2016;7:12623. 27554168 10.1038/ncomms12623 PMC4999515

[7] Zhang Y , Zhang G , Liu Y , et al. GDF15 regulates Malat‐1 circular RNA and inactivates NFkappaB signaling leading to immune tolerogenic DCs for preventing alloimmune rejection in heart transplantation. Front Immunol. 2018;9:2407. 30425709 10.3389/fimmu.2018.02407 PMC6218625

[8] Li M , Zhang X , Zheng X , et al. Immune modulation and tolerance induction by RelB‐silenced dendritic cells through RNA interference. J Immunol. 2007;178(9):5480‐5487. 17442929 10.4049/jimmunol.178.9.5480

[9] Wang B , Zhou Q , Li A , et al. Preventing alloimmune rejection using circular RNA FSCN1‐silenced dendritic cells in heart transplantation. J Heart Lung Transplant. 2021;40(7):584‐594. 34052126 10.1016/j.healun.2021.03.025

[10] Zhang Y , Moszczynski LA , Liu Q , et al. Over‐expression of growth differentiation factor 15 (GDF15) preventing cold ischemia reperfusion (I/R) injury in heart transplantation through Foxo3a signaling. Oncotarget. 2017;8(22):36531‐36544. 28388574 10.18632/oncotarget.16607 PMC5482674

[11] Altesha MA , Ni T , Khan A , Liu K , Zheng X . Circular RNA in cardiovascular disease. J Cell Physiol. 2019;234(5):5588‐5600. 30341894 10.1002/jcp.27384

[12] Li A , Wang WC , McAlister V , Zhou Q , Zheng X . Circular RNA in colorectal cancer. J Cell Mol Med. 2021;25(8):3667‐3679. 33687140 10.1111/jcmm.16380 PMC8051750

[13] Gokool A , Anwar F , Voineagu I . The landscape of circular RNA expression in the human brain. Biol Psychiatry. 2020;87(3):294‐304. 31570194 10.1016/j.biopsych.2019.07.029

[14] Xu T , Wu J , Han P , Zhao Z , Song X . Circular RNA expression profiles and features in human tissues: a study using RNA‐seq data. BMC Genomics. 2017;18(Suppl 6):680. 28984197 10.1186/s12864-017-4029-3 PMC5629547

[15] Du WW , Yang W , Li X , et al. A circular RNA circ‐DNMT1 enhances breast cancer progression by activating autophagy. Oncogene. 2018;37(44):5829‐5842. 29973691 10.1038/s41388-018-0369-y

[16] Chen Q , Mang G , Wu J , et al. Circular RNA circSnx5 controls immunogenicity of dendritic cells through the miR‐544/SOCS1 Axis and PU.1 activity regulation. Mol Ther. 2020;28:2503‐2518. 32681834 10.1016/j.ymthe.2020.07.001 PMC7646215

[17] Zheng X , Suzuki M , Ichim TE , et al. Treatment of autoimmune arthritis using RNA interference‐modulated dendritic cells. J Immunol. 2010;184(11):6457‐6464. 20435931 10.4049/jimmunol.0901717

[18] Su Y , Zhu C , Wang B , et al. Circular RNA Foxo3 in cardiac ischemia‐reperfusion injury in heart transplantation: a new regulator and target. Am J Transplant. 2021;21(9):2992‐3004. 33382168 10.1111/ajt.16475

[19] Zhu C , Su Y , Juriasingani S , et al. Supplementing preservation solution with mitochondria‐targeted H2 S donor AP39 protects cardiac grafts from prolonged cold ischemia‐reperfusion injury in heart transplantation. Am J Transplant. 2019;19(11):3139‐3148. 31338943 10.1111/ajt.15539

[20] Zheng H , Su Y , Zhu C , et al. An addition of U0126 protecting heart grafts from prolonged cold ischemia‐reperfusion injury in heart transplantation: a new preservation strategy. Transplantation. 2021;105(2):308‐317. 32776778 10.1097/TP.0000000000003402

[21] Li H , Shi B . Tolerogenic dendritic cells and their applications in transplantation. Cell Mol Immunol. 2015;12(1):24‐30. 25109681 10.1038/cmi.2014.52 PMC4654373

[22] Rybak‐Wolf A , Stottmeister C , Glazar P , et al. Circular RNAs in the mammalian brain are highly abundant, conserved, and dynamically expressed. Mol Cell. 2015;58(5):870‐885. 25921068 10.1016/j.molcel.2015.03.027

[23] Ouaaz F , Arron J , Zheng Y , Choi Y , Beg AA . Dendritic cell development and survival require distinct NF‐kappaB subunits. Immunity. 2002;16(2):257‐270. 11869686 10.1016/s1074-7613(02)00272-8

[24] Sica A , Dorman L , Viggiano V , et al. Interaction of NF‐kappaB and NFAT with the interferon‐gamma promoter. J Biol Chem. 1997;272(48):30412‐30420. 9374532 10.1074/jbc.272.48.30412

[25] Lai KS , Jin Y , Graham DK , Witthuhn BA , Ihle JN , Liu ET . A kinase‐deficient splice variant of the human JAK3 is expressed in hematopoietic and epithelial cancer cells. J Biol Chem. 1995;270(42):25028‐25036. 7559633 10.1074/jbc.270.42.25028

[26] Long X , Zhao B , Lu W , et al. The critical roles of the SUMO‐specific protease SENP3 in human diseases and clinical implications. Front Physiol. 2020;11:558220. 33192553 10.3389/fphys.2020.558220 PMC7662461

[27] Hu Z , Teng XL , Zhang T , et al. SENP3 senses oxidative stress to facilitate STING‐dependent dendritic cell antitumor function. Mol Cell. 2021;81(5):940‐952 e945. 33434504 10.1016/j.molcel.2020.12.024

[28] Williams JA , Tai X , Hodes RJ . CD28‐CD80/86 and CD40‐CD40L interactions promote thymic tolerance by regulating medullary epithelial cell and thymocyte development. Crit Rev Immunol. 2015;35(1):59‐76. 25746048 10.1615/critrevimmunol.2015012501 PMC7711338

[29] Sakaguchi S , Yamaguchi T , Nomura T , Ono M . Regulatory T cells and immune tolerance. Cell. 2008;133(5):775‐787. 18510923 10.1016/j.cell.2008.05.009

[30] Roncarolo MG , Levings MK , Traversari C . Differentiation of T regulatory cells by immature dendritic cells. J Exp Med. 2001;193(2):F5‐F9. 11208869 10.1084/jem.193.2.f5 PMC2193342

[31] Raker VK , Domogalla MP , Steinbrink K . Tolerogenic dendritic cells for regulatory T cell induction in man. Front Immunol. 2015;6:569. 26617604 10.3389/fimmu.2015.00569 PMC4638142

[32] Giannoukakis N , Trucco M . Dendritic cell therapy for type 1 diabetes suppression. Immunotherapy. 2012;4(10):1063‐1074. 23148758 10.2217/imt.12.76

[33] Morante‐Palacios O , Fondelli F , Ballestar E , Martinez‐Caceres EM . Tolerogenic dendritic cells in autoimmunity and inflammatory diseases. Trends Immunol. 2021;42(1):59‐75. 33293219 10.1016/j.it.2020.11.001

[34] Zheng X , Suzuki M , Zhang X , et al. RNAi‐mediated CD40‐CD154 interruption promotes tolerance in autoimmune arthritis. Arthritis Res Ther. 2010;12(1):R13. 20102615 10.1186/ar2914 PMC2875641

