# Supplementary material for: Circular RNA MAP2K2‐modified immunosuppressive dendritic cells for preventing alloimmune rejection in organ transplantation
Source: Bioeng Transl Med. 2023 Nov 13;9(1):e10615. doi: 10.1002/btm2.10615 (PMC10771550; doi:10.1002/btm2.10615)
Supplement: Supplementary file 1 — TABLE S1. Primer, probe and siRNA sequences. FIGURE S1. circMAP2K2 siRNA did not change cell apoptosis/death. In vitro cultured DCs were transfected with circMAP2K2 siRNA or control GL2 siRNA for 48 h. Cells were then collected and stained with fluorescent APC‐Annexin V and PI (Therom Fisher Scientific) according to the manufacturer's instruction. The intensity of fluorescence was measured using a Cytoflex S (Beckman). Left: Representative graphs of dot plot results; Right: Summarized data of Annexin V positive cell percentage. FIGURE S2. Knockdown of circMAP2K2 in DCS enhanced CD4+ CD25+ Foxp3+ cell production. CD4+CD25− T cells were isolated from naïve Babl/c mice using a CD4CD25 T cell isolation kit ((Miltenyi Biotec, San Jose, CA) following the manufacturer's instruction. Isolated CD4+CD25− T cells were co‐cultured with circMAP2K2 siRNA or GL2 siRNA transfected DCs at the ratio of 1:10 for 5–7 days. [file BTM2-9-e10615-s001.docx]

**Supplemental methods**

**1. DC culture**

Bone marrow-derived DCs (BM-DCs) were cultured from bone marrow progenitor cells with RPMI with RPMI-1640 medium (Thermo Fisher Scientific, Mississauga, Ontario, Canada) in the presence of 10% fetal bovine serum (FBS, Thermo Fisher Scientific), 10 ng/ml of granulocyte-macrophage colony-stimulating factor (GM-CSF, PeproTech, Rochy Hill, NJ) and 10 ng/ml of interleukin 4 (IL-4, PeproTech), and 100 U penicillin and streptomycin (Thermo Fisher). Half of medium was replaced every other days. ^11,18^

**2. DC transfection with siRNA**

On day 5 of cell culture, BM-DCs (1 million/well in a 12 well plate) were transfected with 1 µg siRNA using 2 µl EndoFectin™ (Gene Copoeia Inc., Rockville, MD) in 400 µl DC-medium without antibiotics at 37°C and 5% CO_2._ Both circMAP2K2 siRNA (sense: G UCAGCA UUGCG GGCAA ACCTG GT) and Firefly luciferase GL2 siRNA were purchased from Sigma-Aldrich (Sigma-Aldrich, Mississauga, Canada). GL2 siRNA was used as control siRNA Another 500 µl DC-medium was then added to the cells and cells continued to culture. On the next day of transfection, another 1 ml fresh DC-medium was added to each well. 48 h after transfection, DCs were harvested for the subsequent experiments. In some experiments cy3-labelled circMAP2K2 siRNA (Sigma) was used for imaging cells after transfection.

**3. DC staining and flow cytometry**

DCs were collected and resuspended at 2x10^6^ cells/mL in cell culture medium. 2x10^5^ cells were taken and incubated with fluorescent CD11c-FITC, CD40-PE-cy5, CD80-PE and MHCII-fluor 450 (Biolegend, San Diego, CA) for 15 minutes at room temperature in the dark. Cells were then washed with 1 mL of PBS containing 2% FBS, centrifuged at 300g for 10 min, and resuspended in PBS containing 2% FBS. Samples were run on a Cytoflex S (Beckman coulter, Indianapolis, IN) for flow cytometry analysis. Mean fluorescence intensity (MFI) was collected and compared between groups

**4. cDNA synthesis and qPCR**

cDNA is generated using an ABM OneScript Plus cDNA synthesis kit (Applied Biological Materials Inc (ABM), Richmond, BC, CANADA) with random primers according to manufacturer’s protocol. Briefly, 2 µg of total RNA was mixed with 1 µL random hexamer (10 µM), 1 µL dNTP (10 µM) and diluted to 15 µL. Samples were incubated at 65°C for 5 min followed by ice for 2 min. 4 µL 5x buffer and 1 uL reverse transcriptase were added and samples were incubated at 25°C for10 min, 55°C for 15 min and 85°C for 5 min in a T100 Thermal Cycler (BioRad, Mississauga, Canada). The resulting cDNA was diluted 10 times with ultra-pure water (Thermol Fisher Scintific) for quantitative polymerase chain reaction (qPCR).

qPCR was executed with Brightgreen SYRB green mixture (ABM) with 300 nM of the forward and reverse primers listed in Supplementary Table 1 and 2 µl of the 1:10 diluted cDNA. Reactions were run on a CFX connect real-time PCR system (Bio-Rad Laboratories) using 95°C for 10 min, followed by 40 cycles of 95°C for 15s, 60°C for 1 min and a melt curve. Relative quantification was calculated using the 2^-ΔΔ Ct^ method. β-actin or GAPDH was used as a loading control.

**5. Protein isolation and Western Blotting**

Total protein was directly lysed from cells with 1x RIPA with protease inhibitors PFMC or isolated from Trizol after the aqueous layer was taken for RNA according to the manufacturer’s manual. Briefly, after aqueous layer removal from Trizol-chloroform, 1.5 mL isopropanol was added per 1 mL Trizol and incubated for 10 min at room temp (RT) prior to centrifugation at 12,000g at 4°C for 10 min. The supernatant was discarded and the protein pellet washed twice with 1 mL 80% cold ethanol at RT for 20 min, followed by centrifugation at 7,500g 4°C for 5 min. The pellet was air dried and resuspended in 1% SDS at 55°C for 1 h. Samples were spun down again 10,000g 4°C for 10 min and the supernatant containing protein was collected and concentration measured using a BAC kit.

15-25 µg of total protein was mixed with loading buffer, boiled at 95°C for 5 min and chilled on ice for 2 min. Samples were loaded into a 12% SDS- polyacrylamide gel, run at 60V for 30 min and 100V for 90 min, and then transferred onto a PVDF membrane (Bio-Rad Laboratories) by a Trans-blot turbo system (BioRad laboratories) according to the manufacturer’s protocol. Membranes were blocked with 5% Fat-free milk in 0.2% PBS-Tween 20 (PBST) for 1 h at RT and then blotted overnight at 4°C with primary antibodies against: SENP3(Santa Cruz, 1:1500), p-p65(Cell Signaling Technology (CST) Whitby, Ontario, Canada,1:2000), p65(CST, 1:2000), MEK2 (CST 1:2000), Erk1/2 and P-ERK1/2 (CST, 1:2000) and β-actin (Santa Cruz, 1:4000). Membranes were washed 3 x for 10 min in PBST and incubated for 2 h at RT with appropriate secondary antibody (1:4000). Membranes were then washed 3x 10 min with PBST again prior to exposure with clarity max ECL (Bio-Rad laboratory) and visualized using a FluorChem M system (ProteinSimple, San Jose, CA). Densitometry was done using ImageJ to determine relative expression and samples were normalized to their respective B-actin as a loading control.

**6. Immunocytochemistry**

Cover slips were pre-coated with 1% gelatin for 2h in a 24-well plate, followed by plating of 40000 DCs suspended in CM and overnight incubation. Cells were washed with PBS and fixed with 4% PFA for 15 min at RT, followed by washing with PBS three times. Cells were permeabilized with 0.25% Triton X-100 in PBS for 10 min at RT and washed again three times with PBS. Cells were blocked with 3% BSA in PBS for 30 min at RT prior to antibody exposure. Cells were blotted with primary Abs overnight at 4°C (SEBP3, and P-p65 Abs, 1:200), washed three times with PBS and subjected to appropriate secondary antibody containing Fluor-Alexa 488 fluorophore (1:250) at RT for 2 h in the dark. Cells were washed three time in PBS, incubated with 1:10,000 DAPI for 5 min, rinsed with PBS and imaged under a T-92 eclipse microscope.

**7. RNA immunoprecipitation Assays and Co-IP**

Day 8 cultured DCs were treated with 100ng/mL LPS for 2 h at 37°C to stimulate maturation which was validated via DC phenotype as described above. 10 x10^6^ cells were collected and lysed with 1x RIPA buffer (cell signaling) containing PMSF (cell signaling) on ice for 20 min and centrifuged at x 12,000g at 4°C for 20 min and supernatant was collected. The concentration of protein in the supernatant was measured by the Bradford assay (Bio-Rad).

For RIP, cell supernatant containing 500-1000 µg of protein was mixed with 3 µg of denatured biotinylated DNA probes with a total volume of 200 µL and incubated at RT for 2 h on a rotator. During this time, streptavidin dynabeads (ThermoFisher) were prepared for RNA samples according to the manufacturer’s protocol. After 2 h, 50 µL of prewashed streptavidin beads was added to each sample and incubated for 1 h at RT. Samples were then washed 6 times with 200 µL of RIPA in a magnetic field. Samples were then subjected to Mass Spectromety, or resuspended in 20 µL ultra-pure and heated at 65°C for 10 min to elute immunoprecipitate. 200 µL TRIzol was added and the RNA was isolated using a miRNeasy kit (Qiagen, San Diego, CA) according to the manufacturer’s instructions and used for cDNA synthesis as described above.

For Co-IP, 2 µg of SENP3 Abs, or control IgG, were mixed/1 mg total protein and incubated on a rotator overnight at 4°C. 18 µl of protein A/G agarose beads was then added into each sample and incubated at RT for 2 h. Samples were spun at 2000 g 4°C for 5 min, supernatant decanted and the beads were washed with 1 mL 1x RIPA buffer. Wash was repeated 5 times. Beads were then put into 200 µL TRIzol for RNA/protein separation as described above.

**Supplemental Table 1 Primer, probe and siRNA sequences**

| Gene or oligo | Forward Sequence | Reverse Sequence |
| --- | --- | --- |
| circMAP2K2 | AGTGCAACTCGCCCTACATC | CACCAGGTTTGCCCGCAATG |
| MAP2K2/MEK2 | TGTAGCTGAGCTGTGGGTAG | ATCTTGCAGCCGGGAAAGAG |
| β-actin | CGTGAAAAGATGACCCAGATCA | CACAGCCTGGATGGCTACGT |
| CCR7 | AGACCACCACAACCTTCTCC | ACTGGCCAGAATTGCTCTTC |
| GAPDH | GGGGTGAGGCCGGTGCTGAGTAT | CATTGGGGTAGGAACACGGAAGG |
| IL-1 | AACCCAGATCAGCACCTTACACCT | TGGCAACTCCTTCAGCAACAC |
| IL-6 | CGTGGAAATGAGAAAAGAGTTGTG | TGGAAATTGGGGTAGGAAGGA |
| IL-12 | ATGATGACCCTGTGCCTTGG | CCTTTGGGGAGATGAGATGT |
| IL-23 | GCAACTCTGACTGAGCCCTT | CTTGCCCTTCACGCAAAACA |
| circMAP2K2 probe template | CAGCATTGCGGGCAAACCTGCAGCATTGCGGGCAAACCTGCAGCATTGCGGGCAAACCTG | |
| circMAP2K2 probe primer | CAGCATTGCGGGCA | CTGTCAGGTTTGCC |
| Random probe template | ATGCAATGGCACGTACGAGCGATCTATGCCGAATGCAATGGCACGTACGAGCGATCTATGCCGA | |
| Random probe primer | ATGCAATGGCACGTAC | ACGGCATAGATCGCTC |
| circMAP2K2 siRNA sense | GUCAGCAUUGCGGGCAAACCUGGU | |

**Supplementary Figures**

**
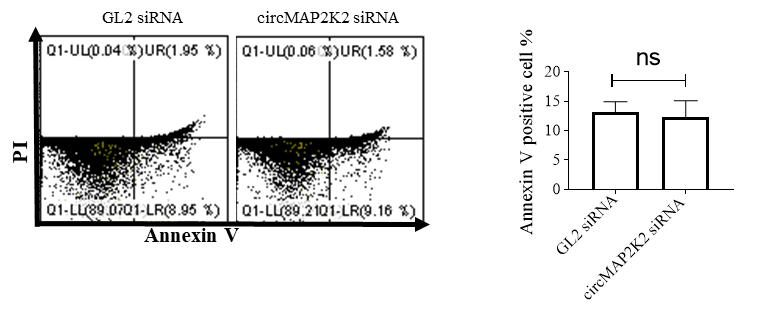
**

**Supplementary Figure S1 circMAP2K2 siRNA did not change cell apoptosis/death.** In vitro cultured DCs were transfected with circMAP2J2 siRNA or control GL2 siRNA for 48h. Cells were then collected and stained with fluorescent APC-Annexin V and PI (Therom Fisher) according to the manufacturer’s instruction. The intensity of fluorescence was measured using a Cytoflex S (Beckman). Left: Representative graphs of dot plot results; Right: Summarized data of Annexin V positive cell percentage.


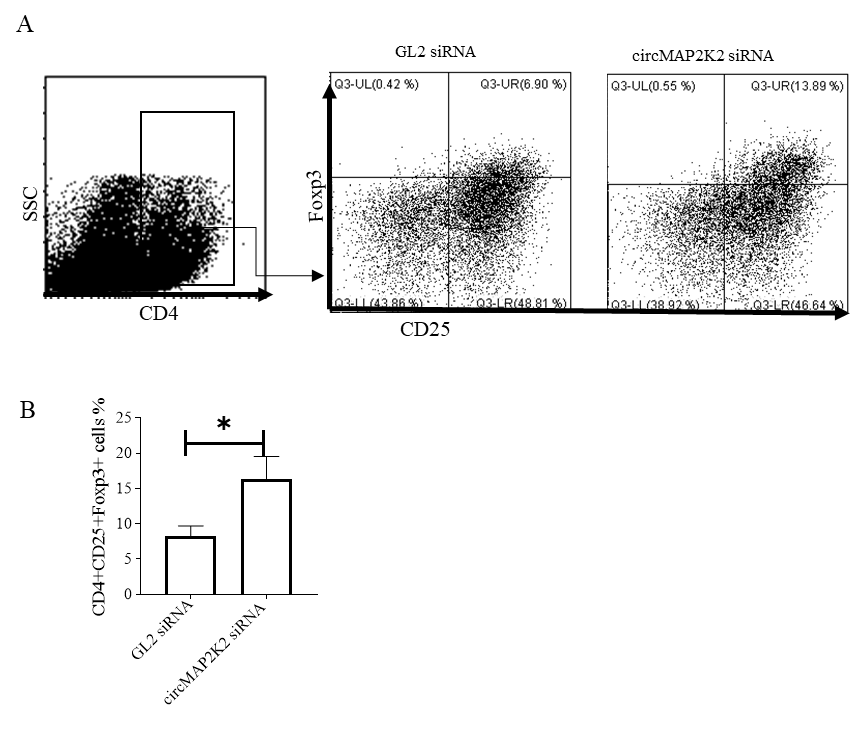


**Supplementary Figure S2 Knockdown of circMAP2K2 in DCS enhanced CD4+CD25+ Foxp3+ cell production**. CD4^+^CD25^-^ T cells were isolated from naïve Babl/c mice using a CD4CD25 T cell isolation kit ((Miltenyi Biotec, San Jose, CA) following the manufacturer’s instruction. Isolated CD4^+^CD25^-^ T cells were co-cultured with circMAP2K2 siRNA or GL2 siRNA transfected DCs at the ratio of 1:10 for 5-7 days.

Cells were then stained with fluorescent CD4, CD25 and Foxp3 Abs using a Treg staining kit (Therom Fisher), followed by flow cytometry using a Cytoflex S (Beckman)
